# Street Sweeping by Skirts

**Published:** 1901-08-24

**Authors:** 


### The Hospital

A Journal of

The Medical Sciences and Hospital Administration.

Vol. XXX.—No. 778.] Edited by Sir Henry Burdett, K.C.B., and Solomon C. Smith, M.D., M.R.C.P. [August 24, 1901.

### Street Sweeping by Skirts.

The inexpediency of permitting or requiring ladies to accomplish, by the agency of their trailing skirts, a sweeping of the footpaths analogous to that which is done for the roadways by revolving brooms, and the consideration of the various evils which may arise from the custom, has for a considerable time furnished an useful, if not an agreeable, topic for professional journalists and for amateur correspondents. Until the present year, this topic was considered to have its appropriate period of appearance in that which our ancestors were wont to describe as "the silly season," but it has lately been elevated, by the proceedings of the Congress on Tuberculosis, into a position in which it may claim at least a parity with the eloquence of Irish members of the House of Commons, and may be discussed, with or without advantage, even within the sacred limits of the parliamentary session itself. Of this altered position of the question we have not so far availed ourselves; but it must not be supposed that our silence has proceeded from indifference. We have long been studious observers of the varied and beautiful cycloidal curves into which the dust of the footpaths is arranged by the skirts of female pedestrians, and have often pondered on the course and destination of the less weighty particles, and on the consequent bacterial flora and general cleanliness of what would be called, in America, the "lower extremities" of the fair sweepers. We have even thought about the amplitude and the radii of the curves themselves; and have speculated on the extent to which these might afford indications of the stature, the character, or even the personal appearance, of the producer. All varieties are to be seen, from the bold and far extending swing produced by a stride of almost masculine extent and energy, down to the niggling curves left by a lady whose tight or misfitting boots fail to afford her even an approach to natural freedom of movement. Occasionally, at least, what may be described as a sporting interest attaches itself to the performance, as the advancing skirt annexes and discards the various objects which the pavement may present. We lately watched the gradual conveyance, from the neighbourhood of Regent Circus, Oxford Street, to the door of the Langham Hotel, of an inexpressibly filthy piece of newspaper, covered with grime and saturated with grease, which had probably been used to wrap up the broken victuals of some tramp, and had been discarded by him when that office had been fulfilled. No less than five skirts were required for the indicated journey; and we much regretted that a more important engagement prevented us from devoting an hour or so to the further travels of the derelict. It would be interesting to know whether it is still a wanderer, or whether it found a final refuge in the wardrobe of the last lady to whom it clung.

With the single exception of Lady Harberton, none of the correspondents whose letters we have seen in print have gone so far as to suggest any practicable course for the initiation of reform, and her ladyship's "rational dress" must, we fear, still be regarded as somewhat in advance of the wishes of the majority of her contemporaries. We are told that the long skirts are too graceful and elegant to be abandoned, but surely their grace and elegance must be dependent upon their adaptability for the occasions on which they are worn, or at least, as Lord Jeffrey showed to be the case with the beauty of dress in general, upon the circumstance that we associate them with the qualities of those by whom they were first made fashionable. We are prepared to maintain, against all comers, that the English gentlewomen of the eighteenth century have not been surpassed by their descendants in any feminine charm, even if they have been equalled, and although none could have carried Court trains with greater dignity than they, none could have looked prettier or more charming in the short skirts and sandalled shoon with which they smile at us from their pictures. One of Charles Dickens' pet characters is only described as "the young lady with fur round her boots," and it is manifest that such a description in the present day would fail to afford any means of identification. If Bond Street cannot rise to the occasion and invent something in the way of foot covering which ladies will not willingly conceal, surely the ingenuity of Russian bootmakers—the best in the world—might be successfully appealed to. Think, again, of the opportunities which might be afforded to stocking-weavers and embroiderers. It will be objected that we propose to appeal only to vanity and cupidity; and the shrieking sisterhood will tell us that the shortening of skirts should be regarded as a lofty mission to be accomplished by Woman (with a big

W), by reason of her due appreciation of the injurious effects of their prolongation. Be it so. We would refuse no assistance; but we confess to a belief that the vanity and cupidity, if only they could be stirred into full operation, would accomplish more in a month than the more exalted considerations in a year. If we rest upon the latter alone, or upon the transient fizzle of chatter which has followed the proceedings of the Congress, the parody of the "Song of the Shirt," recently published by a contemporary, will long retain its present applicability:—

"Sweep—sweep—sweep—
As we walk o'er the West End flags,
For, however we try to carry that tail,
A part of it always sags—
The hem of it always drops
In the winter's greasy slush;
The hem of it sweeps the summer's dust
More clean than the dustman's brush."

